# Graphene-Based Absorber: Tunable, Highly Sensitive, Six-Frequency

**DOI:** 10.3390/molecules30081688

**Published:** 2025-04-10

**Authors:** Xinmei Wang, Xianding He, Hua Yang, Xu Bao, Yongjian Tang, Pinghui Wu, Yougen Yi

**Affiliations:** 1 Chengdu Aeronautic Polytechnic, School of Unmanned Aerial Vehicles Industry, Chengdu 610100, China; wangxinm1006@163.com (X.W.); hx172336244@163.com (X.H.); 2 State Key Laboratory of Advanced Processing and Recycling of Non-Ferrous Metals, Lanzhou University of Technology, Lanzhou 730050, China; hyang@lut.cn; 3 Joint Laboratory for Extreme Conditions Matter Properties, Southwest University of Science and Technology, Mianyang 621010, China; baoxu0409@163.com (X.B.); tangyongjian2000@sina.com (Y.T.); 4 Fujian Provincial Key Laboratory for Advanced Micro-Nano Photonics Technology and Devices & Key Laboratory of Information Functional Material for Fujian Higher Education, Quanzhou Normal University, Quanzhou 362000, China; phwu@zju.edu.cn; 5 College of Physics, Central South University, Changsha 410083, China

**Keywords:** six-frequency absorber, insensitive incidence angle, insensitive polarization, high sensitivity

## Abstract

Due to the equipartition exciton property of graphene metamaterials, researchers have applied them to the design of absorbers and developed a series of absorbers covering different wavebands (including narrowband and broadband). In this paper, an absorber based on surface-isotropic excitations was designed with the help of graphene metamaterials and relevant simulations. The absorber exhibited six perfect absorption peaks in the mid-infrared band and had an extremely simple structure consisting of only three layers: a gold layer at the bottom, a dielectric layer made of silica in the middle, and patterned graphene at the top. This absorber possesses excellent tuning ability, and by applying an external bias to the graphene layer, the Fermi energy level of graphene can be adjusted, and thus the resonance frequency of the absorption peak can be tuned. Meanwhile, the effect of the graphene relaxation time on the absorber performance was investigated. In addition, the refractive index of the dielectric layer was found to be linearly related to the resonance frequency of the absorption peak. It is worth mentioning that the absorber structure possessed polarization insensitivity due to its central symmetry. Even when incident light with different polarizations was incident over a wide range of angles, the change in absorbance of the absorption peaks was negligible, demonstrating significant insensitivity to the angle of incidence. The sensor possesses excellent characteristics such as tunability, polarization insensitivity, incident angle insensitivity, and high sensitivity. This paper demonstrates the feasibility of a six-frequency sensor and opens up more ideas for the design of multi-frequency sensors.

## 1. Introduction

The progress of human civilization is closely linked to the development of tools, which in turn depends on the innovation of materials. As people’s pursuit of a better life continues to rise, there is a growing demand for materials with excellent performance and wide applicability. In the information age, there is a critical need for the regulation, absorption, and transformation of electromagnetic waves [1,2,3]. For example, information transmission uses electromagnetic waves as a carrier, radar stealth needs to absorb electromagnetic waves, and photovoltaic power generation requires the energy conversion of electromagnetic waves [4,5]. Among them, the research on optical absorbers has become a key area, and its applications cover many aspects such as sensing [6], imaging [7], optical switching [8], and so on.

Conventional optical absorbers usually have a three-layer structure, with a continuous metal layer at the bottom, a structure that prevents electromagnetic waves from penetrating the absorber, a dielectric layer in the middle, and a periodically structured metal at the top [9,10,11]. This structure utilizes the equipartition excitations generated on the surface of the metal to confine the electromagnetic waves, thus achieving the perfect absorption of electromagnetic waves. Surface plasmonic excitations (SPs) are electromagnetic modes with a high degree of localization that form when free electrons on a metal surface couple with the incident light when the incident light strikes the surface [12,13,14].

The development of metamaterials has opened up new avenues for the design of optical absorbers. The year 2008 saw the first application of metamaterials to optical absorbers by Landy’s team [15], which resulted in the perfect absorption of absorption peaks and tunable performance, while 2004 saw the first application of graphene metamaterials to optical absorbers by mechanical exfoliation [16] through the persistent efforts of two physicists, K. S. Novoselov and A. K. Geim. Graphene consists of monolayers of C atoms covalently bonded by SP^2^ hybridized electron orbitals and has a hexagonal (honeycomb) structure. As a two-dimensional material, monolayer graphene has a series of excellent properties. Its conduction and valence bands intersect at one point, the Dirac point, where it exhibits a linear dispersion relation. Due to the presence of free large π-bonds in graphene, electrons are able to move freely in the graphene sheet, which gives graphene extremely high electrical and thermal conductivity. However, the absorption of light by a single layer of graphene is about 2.3% [17,18]. In 2006, Vafek et al. [19] predicted the ability of graphene to excite surface-isolated excitations based on a theoretical study. By 2008, Hanson et al. [20] found that graphene had metal-like properties in many respects, and even outperformed metals in some aspects. Compared with metal surface plasmonic excitons, graphene surface plasmonic excitons have more advantages such as lower transmission loss, longer lifetime, convenient tunability, and especially strong localization. In 2012, two independent research groups, Chen and Fei, conducted imaging experiments on graphene, and Chen et al. [21] investigated the propagation and propagation of surface plasmonic excitons on graphene by scanning near-field microscopy. Tang et al. [22] investigated the propagation and localization properties of equipartitioned excitations on the surface of graphene by scanning near-field microscopy. With the introduction of graphene metamaterials, new ideas for designing high-performance optical absorbers have been proposed. Subsequent researchers have utilized the property of equipartitioned excitons on the surface of graphene to design many absorbers with narrowband [23,24] and broadband [25,26] characteristics.

The basic structural unit of the absorber studied in this paper consisted of a continuous gold layer as the bottom layer, silica as the dielectric layer, and a top layer consisting of four concave-structured graphenes. The model takes full advantage of graphene’s localized surface equipartition excitations in the mid-infrared band to achieve the excellent performance of six perfect absorption peaks. The absorptivities of its six absorption peaks were 0.9872, 0.9836, 0.9803, 0.9931, 0.9965, and 0.9870, respectively. The absorption mechanism of this absorber can be explained by observing the electric field distribution on its surface. Meanwhile, the Fermi energy levels of graphene were controlled by bias voltage, and their effects on the absorption spectra were investigated by simulating the change in the relaxation time of graphene. It was found that the change in the refractive index of the intermediate electric layer of the absorber induced a change in the resonance frequency of the absorption peak. In addition, the scope of application of the absorber in terms of light polarization and incident light incidence angle was investigated. Moreover, the sensing characteristics of the absorber were explored by changing the ambient refractive index.

## 2. Results and Discussion

**Figure 1:**
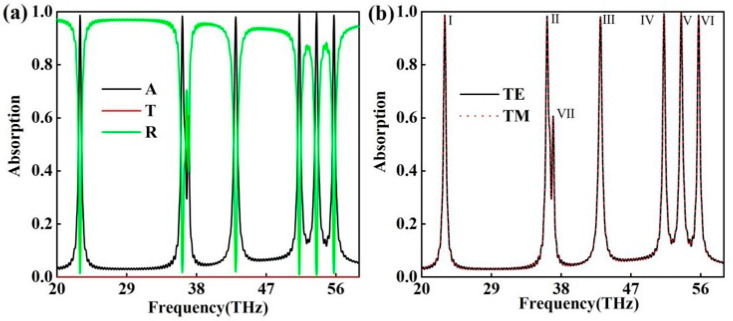
(**a**) The absorption/transmission/reflection spectrum of the absorber. (**b**) The incident light is the TE (transverse electric) wave and the TM (transverse magnetic) wave, and the absorption spectrum of the absorber. The solid black line indicates the TE waves and the red dotted line indicates the TM waves.

As shown in Figure 1a, the transmittance was on the order of 10^−9^, which was so small that it was negligible. This is because the thickness of the gold layer was much larger than the skin depth of the electromagnetic wave in the gold element, resulting in the electromagnetic wave not being able to penetrate the gold layer. The absorber showed seven absorption peaks, which were named I, II, III, IV, V, VI, and VII in Figure 1a. The corresponding resonance frequencies from absorption peak I to VII were 22.9729, 36.1962, 43.0849, 51.2742, 53.4902, 55.7543, and 36.967 THz, and the corresponding absorption rates were 0.98717, 0.98364, 0.98027, 0.99311, 0.99646, 0.98704, and 0.60649, respectively. Since the absorption rate of absorption peak VII was lower than 90%, it had limited influence on the performance of the device, and will not be discussed in the following.

As can be seen in Figure 1b, the absorption spectrum of the absorber did not change regardless of the polarization type of the incident light, which was the TE or TM wave. This was due to the central symmetry of the model, which was not present in the non-centrosymmetric models.

**Figure 2:**
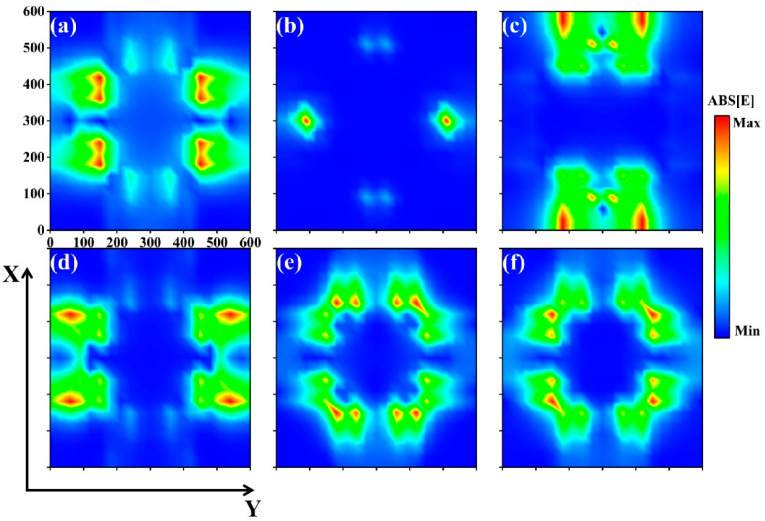
(**a**–**f**) The electric field map at the absorption frequency corresponding to each perfect absorption peak (I)–(VI), with the electric field monitor located at 10 nm above the graphene layer.

As shown in Figure 2, the electric field intensity distribution of the absorber was presented at the resonance frequencies of the six absorption peaks. In absorption peak I, the electric field intensity mainly gathered in the left and right concave protrusion of the edge of the region; in absorption peak II, the electric field intensity was mainly distributed in the left and right concave edge of the region; absorption peak III of the electric field intensity concentrated in the upper and lower concave edge of the two sides of the region and the edge of the concave area; for absorption peak IV, the electric field intensity was located mainly in the left and right concave edge of the two sides of the region; for absorption peak V, the electric field intensity was mainly distributed in the region close to the protrusion of the upper and lower concave zigzags and the protrusion of the left and right concave zigzags [27,28]; and the electric field intensity of absorption peak VI was roughly distributed in the diagonally flipped symmetry. The electric field intensity of absorption peak IV is mainly located in the edge area on both sides of the concave zigzag.

It can be seen from the electric field intensity distribution map that the absorption position of the absorber was mainly concentrated at the edge of the graphene, especially at the edge of the adjacent graphene, and the right-angle position of the four-concave structures was particularly significant. It is precisely because the four-concave structure and size stimulated the surface plasmons of graphene [29,30], which caused the free electrons on the surface of graphene to undergo collective resonance, so the seven absorption peaks of the absorber (six of the absorption peaks for perfect absorption) formed. Among them, the position of the local electric field enhancement of absorption peak VII was similar to that of absorption peak II, but its concentration was not as high as absorption peak II, so it is speculated that because the free electrons produced collective resonance at absorption peak II, the electromagnetic wave at the resonance edge was excited, producing a collective resonance of small amplitude.

**Figure 3:**
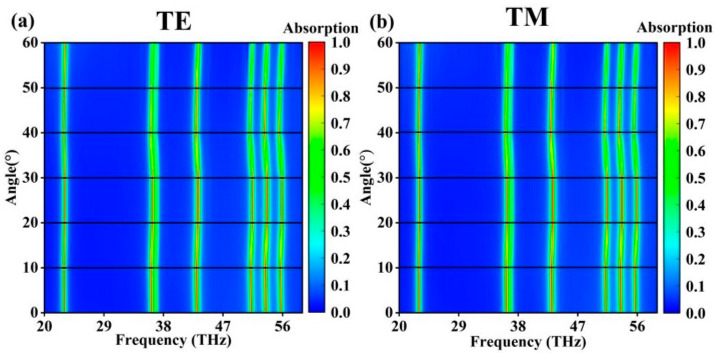
Angle scan. (**a**) TE wave; (**b**) TM wave.

As shown in Figure 3, the insensitivity of the absorber to the angle of incidence is critical in practical applications. In real-world environments, absorbers are difficult to place in ideal optical conditions. The angular scanning plot starts at 0 degrees and scans the angle of incidence in 10-degree steps. It can be seen that in the case of TE waves, the absorption peaks in the absorption spectrum remained high at over 90% absorption when the incident angle was in the range of 0–50°. The angular scan plot for TM waves was highly similar to that for the TE waves. At the same time, the resonance frequency of the absorption peaks changed slightly as the angle of incidence increased. Based on this characteristic, the absorber can be used in applications such as the angular calibration of line light sources, which shows good prospects in reality [31,32,33].

Previously, we explained the absorption mechanism of the absorber based on the electric field distribution of the absorber at the resonance frequency of the absorption peak and investigated the effect of the incident angle of an external light source on the absorber. The follow-up study will focus on the intrinsic properties of the absorber and investigate the effects on the absorption spectrum by changing the Fermi energy level, relaxation time, and refractive index of the dielectric layer of graphene [34,35].

The specific expressions for the Fermi level and the relaxation time of graphene are as follows.

The external DC (direct current) bias voltage $V_{g}$, applied to the graphene layer by the ion gel, can dynamically tune the Fermi level of the graphene [36]. These connections are related as follows:

(1) $$E_{F}=V_{F}\sqrt{\pi \varepsilon_{0}\varepsilon_{r}V_{g}/et_{s}}$$

where $V_{g}$ is the DC bias, $V_{F}$ (10^6^ m/s) is the Fermi velocity, and $t_{s}$ is the dielectric layer thickness. The relaxation time of graphene can be expressed using the following equation [37]:

(2) $$\tau =\mu_{c}\upsilon /eV_{F}^{2}$$

where μ_c_ and υ are the chemical potential and carrier mobility of graphene, respectively.

**Figure 4:**
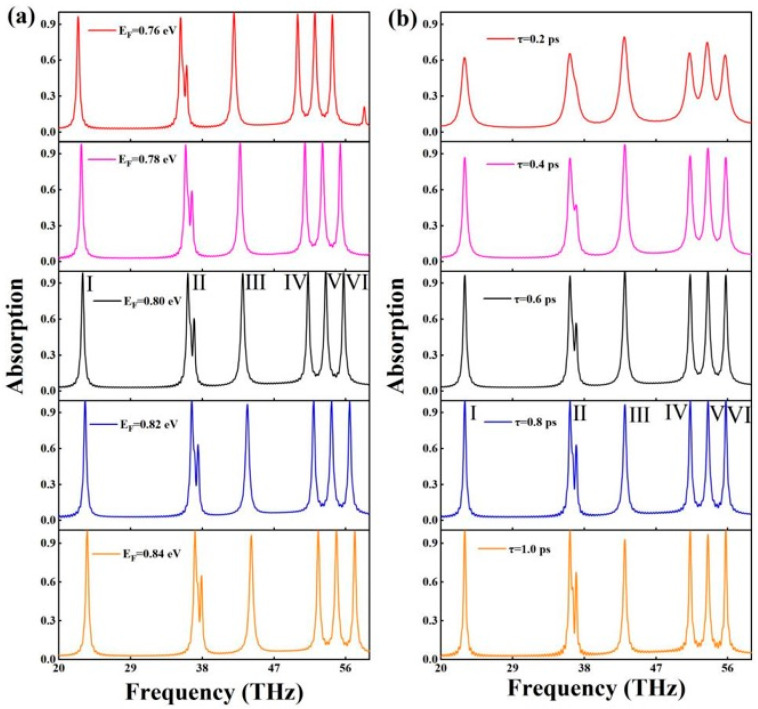
(**a**) Absorption spectrum of the absorber at different Fermi levels of graphene, and (**b**) absorption spectrum of the absorber at differentrelaxation time of graphene.

As shown in Figure 4a, the effect of the graphene Fermi energy levels on the absorption spectrum of the absorber can be clearly visualized. The variation in the key parameter, the Fermi energy level, is particularly important for the absorption spectra when the performance characteristics of the absorber are explored in depth. As the Fermi energy level of graphene rises gradually, the resonance frequency of the absorption peaks increases steadily, and this remarkable phenomenon is explicitly referred to as the blueshift of the absorption peaks in the field of optics [38,39]. Analyzing from the essential principle level, the increase in the Fermi energy level will prompt the excitation process of non-equilibrium carriers inside graphene. The excitation of these non-equilibrium carriers requires high-frequency electromagnetic waves of a specific frequency as energy input. Only when the frequency of the electromagnetic wave meets certain conditions can it effectively provide enough energy for the excitation of the carriers [40,41].

From the perspective of the absorption peak absorption rate, a detailed analysis was carried out using a series of experimental tests and data comparison that found that changing the Fermi energy level of graphene on the absorption peak absorption rate of the impact was extremely small, almost negligible. This experimental result fully demonstrates that the absorption peaks showed a significant insensitivity in the process of changing the Fermi energy level within a certain range. In order to present this property more intuitively, we illustrated it through specific data. When the Fermi energy level of graphene was gradually increased from 0.76 to 0.84 eV, the resonance frequency of each absorption peak showed a significant upward trend. The specific data were as follows: the resonance frequency of the first absorption peak increased steadily from 22.4189 to 23.5509 THz; the second absorption peak increased from 35.281 to 37.0874 THz; the third absorption peak increased from 41.9769 to 44.1447 THz; the fourth absorption peak increased from 49.9736 to 52.5508 THz; the fourth absorption peak increased from 49.9736 to 52.5508 THz; the fifth peak from 52.1414 to 54.839 THz; and the sixth peak from 54.3332 to 57.1272 THz. Through in-depth analysis of these data, it was also found that there is no simple linear relationship between the resonance frequency of the absorption peaks and the Fermi energy level, and the trend of its change showed a more complex disordered state.

Previously, many related studies have clearly shown that the Fermi energy level of graphene can be flexibly regulated by external bias voltage. Based on this close correlation, the absorber has a unique electrical tuning capability, which allows the absorber to be used in a wider range of practical applications [42,43,44].

The correlation between the absorption spectra of this absorber and the relaxation time of graphene is demonstrated in Figure 4b. As can be seen from Figure 4b, the relaxation time of graphene had relatively little effect on the resonance wavelengths of the absorption peaks, but had a significant effect on the absorbance of the absorption peaks. When the relaxation time was 0.2 ps, the absorption rate of most absorption peaks was about 60%, and absorption peaks II and VII were combined into one absorption peak. With the increase in the relaxation time, absorption peaks II and VII gradually separated. In the relaxation time interval from 0.4 to 1 ps, the absorption rates of the perfect absorption peaks showed significant changes, and the specific data were as follows: the absorption rate of absorption peak I changed from 0.86874 to 0.99596; absorption peak II changed from 0.86458 to 0.99916; absorption peak III changed from 0.92768 to 0.99645; absorption peak IV changed from 0.88523 to 0.99779; absorption peak VII changed from 0.88523 to 0.99779; and absorption peak VIII changed from 0.99779 to 0.99645. The above results show that the relaxation time of graphene plays a crucial role in the absorption performance of the absorber.

**Figure 5:**
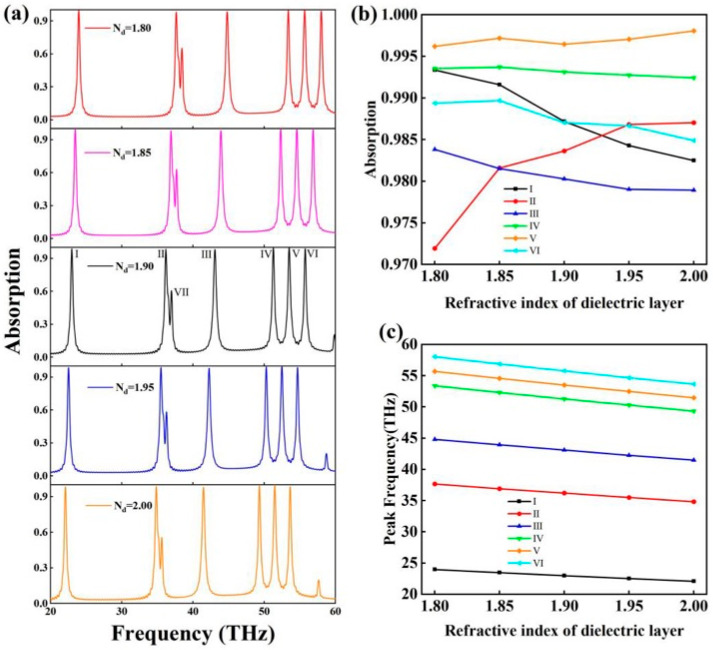
In the case of the refractive index of different dielectric layers, (**a**) the absorption spectrum of the absorber; (**b**) the change in the absorption rate of each absorption peak; and (**c**) the change in the resonance frequency of each absorption peak.

Figure 5a shows the effect of the refractive index of the dielectric layer SiO_2_ on the absorption spectrum of the absorber. As shown in Figure 5b, the effect of the refractive index of the dielectric layer on the absorbance of the absorption peak was disordered and small, and the variation range of each absorption peak was as follows: I—0.9825–0.9934, 1.1%; II—0.9719–0.9870, 1.53%; III—0.9789–0.9838, 0.5%; IV—0.9924–0.9937, 0.13%; V—0.9962–0.9981, 0.19%; VI—0.9849–0.9897, 0.48%). This indicates that the refractive index of the dielectric layer has very little effect on the absorption rate of the absorber. As shown in Figure 5c, the resonance frequency of the absorption peak showed a linear relationship with the refractive index of the dielectric layer. Through the data analysis, the fitting of the refractive index (n) and the resonance frequency (γ) using a linear function was obtained:

(3) $$\gamma_{I}=-9.3935n+40.8206$$

(4) $$\gamma_{II}=-14.211n+63.1971$$

(5) $$\gamma_{III}=-16.6195n+74.6620$$

(6) $$\gamma_{IV}=-20.2325n+89.7160$$

(7) $$\gamma_{V}=-21.0755n+93.5337$$

(8) $$\gamma_{VI}=-21.9185n+97.3995$$

In the above discussion of the refractive index range, the above formula applies. In the previous discussion, the Fermi level of graphene can tune the resonance frequency of the absorption peak, but the analytical expression cannot be obtained due to the complex resonance frequency and the Fermi level of graphene. However, the refractive index of the dielectric layer can linearly tune the resonance frequency of the absorption peak, and the refractive index of the dielectric layer has very little influence on the absorption rate [45,46]. According to the above formula, the absorber can be used to detect changes in the refractive index of the dielectric layer material. Inspired by [47], the material in this paper could change the refractive index of the material through temperature. Using the material of this paper as the dielectric layer material, it was possible to realize the temperature detection, which is only an enlightening effect.

**Figure 6:**
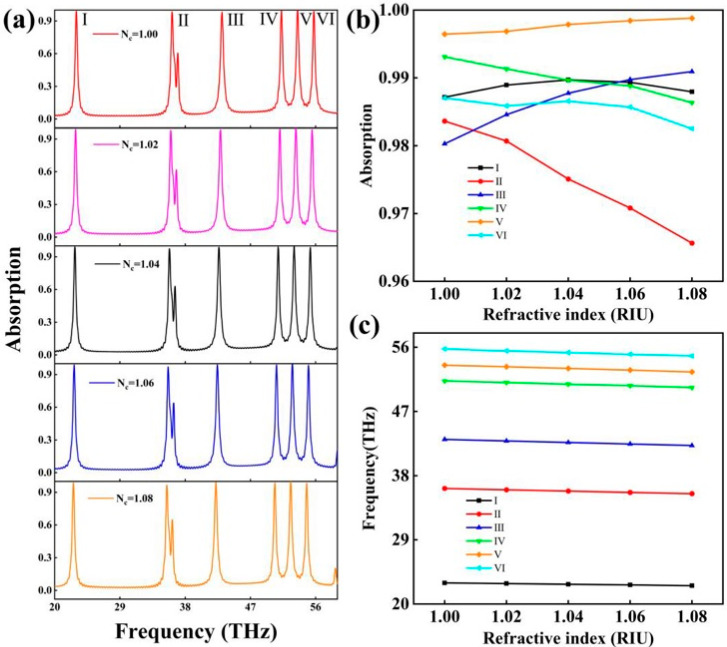
In the case of the different ambient refractive index, (**a**) the absorption spectrum of the absorber; (**b**) the change in the absorption rate of each absorption peak; and (**c**) the change in the resonance frequency of each absorption peak.

Figure 6a shows the influence of the ambient refractive index on the absorption spectrum of the absorber. As can be seen from Figure 6b, the effect of the environmental refractive index on the absorption rate of the absorption rate of the absorption peak was disordered, and the change of the absorption rate was very small, so had little effect on the practical application. However, it can be seen from Figure 6c that the resonance frequency of the absorption peak showed a linear relationship with the ambient refractive index. Through this phenomenon, we can use this performance to detect the atmospheric refractive index as well as in other detection fields. Sensors can measure their sensing performance through the sensitivity (S) and quality factor (FOM) [45,48,49]. The formula of these two indicators is as follows [50,51]:

(9) $$S=\frac{\Delta \lambda }{\Delta n}$$

(10) $$FOM=\frac{S}{FWHM}$$

where Δλ is the amount of change in the resonance wavelength of the absorption peak relative to the change in the ambient refractive index. The FWHM is the half-peak width of the absorption peak. Through the calculation, the S of each absorption peak was 2785.22, 2110.38, 1787.64, 1329.14, 1253.22, and 1212.75 nm/RIU. The absorber had excellent sensitivity for the ambient refractive index. Therefore, the absorber has great prospects for use as a refractive index sensor [52,53,54].

The FOM is an important measure of the absorption peak. To easily calculate the FWHM for all peaks, the background refractive index was set to 1.0. Through calculation, the FOM of each absorption peak was 12.24, 15.86, 21.43, 27.28, 24.59, and 30.42. In sensing applications, high sensitivity is very important for detecting small changes as well as reducing measurement errors and improving measurement accuracy; the quality factor reflects the sensor selectivity, and sensors with a high quality factor have better selectivity for specific measurements and can reduce the influence of other interfering factors.

We used this absorber to compare with recent ones, as shown in Table 1 [55,56,57,58,59]. The absorber of the absorption peak of most and all of the average absorption peaks was relatively high; at the same time, for the incident angle of incidence and polarization sensitivity, the optimal characteristic was the highest sensitivity, but all of the absorption peak FOM were low, the measurement of the measured selectivity was not ideal, and anti-interference ability was weak. Therefore, the absorber can be applied in some special demand areas.

There is some contingency in the design of metamaterial absorbers, so it is impossible to design the metamaterial absorber in demand. Future research directions could introduce mechanical learning to classify the performance of a specific structure, or reverse the design of the absorber of specific demand with the help of a neural network.

## 3. Theory and the Structural Models

**Figure 7:**
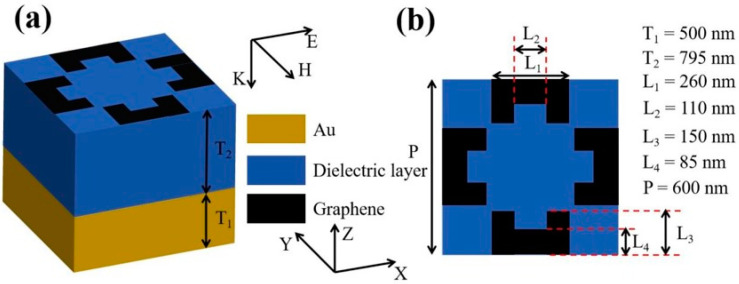
Basic unit of the graphene absorber. (**a**) Stereo diagram. (**b**) Top view.

In this paper, we designed a tunable six-frequency absorber through modeling. The basic unit structure is shown in Figure 7a, where the thickness of the gold layer was T_1_ = 500 nm. The refractive index of the dielectric layer in the middle of the absorber was 1.90, and the thickness of the layer was T_2_ = 795 nm. The top view of the basic unit is shown in Figure 7b. The geometric parameters of the structure were P = 600, L_1_ = 260, L_2_ = 110, L_3_ = 150, and L_4_ = 85 nm, respectively.

The gold material used in this paper was lossy gold, which can be described using the Drude model, and is [60]

(11) $$\varepsilon_{Au}=\varepsilon_{\infty }-\frac{\omega_{p}^{2}}{\omega_{p}^{2}+i\omega \gamma }$$

where $\varepsilon_{\infty }$ = 9.1 is the high-frequency limit dielectric constant. The operating frequency $\omega_{p}$ = 1.3659 × 10^16^ rad/s and the collision frequency γ = 1.0318 × 10^14^ rad/s [61].

The middle layer used a material of SiO_2_ with a refractive index of 1.9. The pattern of the top graphene was a four-concave shape (or two H shape), as shown in Figure 7b. The dielectric constant of the monolayer graphene is [62,63]:

(12) $$\varepsilon \left(\omega \right)=1+\frac{i\delta_{g}}{\omega \varepsilon_{0}\Delta }$$

where δ_g_ is the conductivity of graphene, ω is the angular frequency, $\varepsilon_{0}$ is the vacuum dielectric constant, Δ is the thickness of graphene, and the thickness of graphene is 1 nm.

The conductivity of graphene consists of two parts: intraband conductivity and interband conductivity. Using the kubo formula, the conductivity is [64,65]:

(13) $$\sigma \left(\omega ,\tau ,\mu_{c},Τ\right)=\sigma_{intra}\left(\omega ,\tau ,\mu_{c},Τ\right)+\sigma_{inter}\left(\omega ,\tau ,\mu_{c},Τ\right)$$

(14) $$\sigma_{intra}\left(\omega ,\tau ,\mu_{c},Τ\right)=\frac{2ie^{2}k_{B}T}{\pi \hbar^{2}\left(\omega +i\tau^{-1}\right)}ln\left[2cosh\left(\frac{\mu_{c}}{2k_{B}T}\right)\right]$$

(15) $$\sigma_{inter}\left(\omega ,\tau ,\mu_{c},Τ\right)=\frac{e^{2}}{4\hbar }\left[\frac{1}{2}+\frac{1}{\pi }arctan\left(\frac{\hbar \omega -2\mu_{c}}{2k_{B}T}\right)-\frac{i}{2\pi }ln\frac{{\left(\hbar \omega +2\mu_{c}\right)}^{2}}{{\left(\hbar \omega -2\mu_{c}\right)}^{2}+{\left(2k_{B}T\right)}^{2}}\right]$$

In the formula, ω is the angular frequency, e is the charge of the metacharge, τ is the relaxation time, $\mu_{c}$ is the chemical potential, ℏ is the simple Planck constant, and k_B_ is the Boltzmann constant.

When the frequency of the incident light is low, the energy carried by the light is not enough to induce the electrons to undergo interband transitions, so the interband conductivity is zero in this case [66]. In this case, the conductivity of graphene is only determined by the in-band conductivity, so the Drude model can be used to simplify the conductivity of graphene, and the specific formulas are shown in [67]:

(16) $$\sigma \left(\omega \right)=\frac{ie^{2}\mu_{c}}{\pi \hbar^{2}\left(\omega +i\tau^{-1}\right)}$$

The model was simulated with the help of the FDTD (finite-difference time-domain) module in Ansys Lumerical software (2020 R2). In this paper, the Fermi energy level of graphene was set to 0.80 eV, and the relaxation time was 0.72 ps. In FDTD, the boundary conditions are set as follows: the periodic boundary conditions are used in the x and y directions, the z direction is set as a perfectly matched layer (PML), and the ambient refractive index is set as 1.0 [68,69]. In the present paper, the incident wave was a plane wave, the intensity of the wave was uniformly distributed, the mesh size was 600 nm × 600 nm × 1 nm and set to a graphene layer with a mesh accuracy of 20 nm × 20 nm × 0.5 nm, and the simulation time of 1000 fs. The absorption rate formula for the absorber is quoted from [70,71]:

(17) A=1−R−T

where A is the absorption rate, R the reflectivity, and T the transmission rate because the skin seeking depth of the electromagnetic wave in a good conductor decreases with the increase in the electromagnetic wave frequency, electrical conductivity, and magnetic conductivity of the medium. The thickness of the gold layer we used greatly exceeded the skin seeking depth of the mid-infrared wave, which completely prevented the propagation of the electromagnetic wave, so no light could pass through the gold layer, so T = 0 [72,73]. The absorption rate formula of this absorber can be reduced to

(18) A=1−R

## 4. Conclusions

In this paper, we designed a tunable six-frequency mid-infrared absorber with a top layer consisting of graphene possessing an innovative four-concave structure. By analyzing the electric field distribution on the graphene surface, the field-localized enhancement region of each absorption peak was pointed out. It was found that the increase in the Fermi energy level of graphene induced a blue shift of the absorption peaks; the relaxation time had a decisive influence on the absorbance of the absorption peaks. Coupled with the fact that the Fermi energy levels of graphene could be tuned using a bias voltage, the absorber possessed tunability. The resonant frequency of the absorption peak changed linearly with the refractive index of the intermediate dielectric layer. From the application point of view, the absorber was polarization insensitive, and the absorption peaks maintained a high absorption rate of more than 90% in the range of 0–50° of incident light. At the same time, the high sensitivity of the absorber to the ambient refractive index provides a significant advantage in sensing applications. This paper explores the absorption spectra of four concave-structured graphene absorbers in the mid-infrared wavelength band, which is instructive for concave structures and other absorbers in those four positions.

## Figures and Tables

**Table 1:** Comparison with previous absorbers.

Reference	Peak Number	Average Absorption (%)	Polarization Insensitive	Incident Angle (°)	Sensitivity (nm/RIU)	FOM (1/RIU)
[55]	5	96.47	Yes	0–30	1208.5	-
[56]	3	>99	Yes	0–30	942.6	-
[57]	4	96.78	Yes	0–50	1072.75	182.09
[58]	3	92.34	No	-	2000	188.54
[59]	4	97.99	Yes	0–65	455	111.23
This work	6	98.8	Yes	0–50	2785.22	30.42

## Data Availability

Publicly available datasets were analyzed in this study. These data can be found at https://www.lumerical.com/ (accessed on 1 January 2020).
